# Are physicians aware enough of patient radiation protection? Results from a survey among physicians of Pavia District– Italy

**DOI:** 10.1186/s12913-017-2358-1

**Published:** 2017-06-14

**Authors:** Francesca Campanella, Laura Rossi, Elio Giroletti, Piero Micheletti, Fabio Buzzi, Simona Villani

**Affiliations:** 1 Department of Public Health, Experimental and Forensic Medicine, University of Pavia, Via Forlanini 2, 27100 Pavia, Italy; 2 Department of Physics, University of Pavia, Via Bassi 6, 27100 Pavia, Italy; 3 INFN of Pavia, Via Bassi 6, 27100 Pavia, Italy

**Keywords:** Ionizing radiation, Radiation protection knowledge, Radiological procedures, Physicians’ awareness, Radiation dose

## Abstract

**Background:**

Radiological practices are the first anthropic sources of ionizing radiation exposure of the population. However, a review of recent publications underlines inadequate doctors’ knowledge about doses imparted in medical practices and about patient protection that might explain unnecessary radiological prescriptions. We investigated the knowledge of the physicians of Pavia District (Italy) on the risk of radiation exposure.

**Methods:**

A cross sectional study was performed involving the Medical Association of Pavia District. Data were collected with a self-administered questionnaire, available on-line with private login and password.

**Results:**

Four hundred nineteen physicians fulfilled the questionnaire; 48% of participants reported training about radiation protection. The average percentage of correct answers on the knowledge on ionizing radiation was 62.29%, with a significantly higher result between radiologist. Around 5 and 13% of the responders do not know that, respectively, ultrasonography and magnetic resonance do not expose patients to ionizing radiations. Only 5% of the physicians properly identified the cancer risk rate associated to abdomen computed tomography.

**Conclusions:**

The findings show a quite good level of the general knowledge about ionizing radiations, higher that reported in literature. Nevertheless, we believe the usefulness of training on the risk linked to radiation exposure in medicine for physicians employed in every area.

**Electronic supplementary material:**

The online version of this article (doi:10.1186/s12913-017-2358-1) contains supplementary material, which is available to authorized users.

## Background

In accordance with the estimation of the Italian Agency for the Environmental Protection [1], the effective dose of ionizing radiation from medical sources is about 1.2 mSv/year, while the amount of ionizing radiation from other men made sources is 0.0072 mSv/year. Therefore medical exposure is the first anthropic source of ionizing radiation of the population.

In the last years, the annual number of radiological procedures is exponentially increasing, consequently the collective dose is growing up, especially because of the spread of medical practices imparting non-trivial doses to patients, such as diagnostic Computed Tomography (CT) and interventional radiology. This implies that the risk associated to ionizing radiation, although small at the individual level, might lead to an increased number of cancer cases in the population. The study of Berrington de Gonzales [2] estimated that CT scans performed during 2007 in the USA could be related to about 29,000 future cancers.

Public health policies should support radiation protection culture in medical practice in order to ensure safety cares and the best allocation of available resources [3].

Medical practices involving ionizing radiation are allowed only if justified [4]. The knowledge level on radiation safety of physicians represents a key point to reduce the patient’s exposure in medical practices. Doctors have to know the doses, the detrimental effects associated and the alternatives with lower risk of exposure to avoid the request of unnecessary and unsuitable radiological exams.

Nevertheless, several studies detected lack or poor knowledge about ionizing radiation doses and effects in radiological procedures by the doctors in every specialities and grades.

The aim of the study was therefore to investigate the physicians’ knowledge about patient exposure to ionizing radiation during common radiological procedures and radiation protection awareness.

## Method

### Design and population

All physicians belonging to the Order of Physicians, Surgeons and Dentists of the Pavia District were eligible for the study. Dentists were excluded. As our survey was performed on the entire population of physicians of Pavia District (4410 individuals), is not expected to estimate the sample size.

A cross-sectional design was applied to achieve study aims.

The Ethics Committee of the University of Pavia approved the study protocol.

### Study plan and conduction

The study was carried out from March to May 2013. The board member were invited to attend the study with a letter written by the Order’s Chairman. Furthermore, an advertisement was included on the website of the Medical Association. Precisely, after 1-month from the first invitation, a reminder was sent to the physicians to improve the response rate. To prevent study bias from participant self-education, the actual topic was not clearly mentioned.

At each participant was asked to complete a semi-structured questionnaire of 26 multiple-choice questions concerning radioprotection importance awareness, knowledge about radiation imaging doses and deterministic/stochastic effects, education programs and demographical data (see Additional file 1). Correct answers are based on data published in the Annual Report of the Italian Agency for the Environmental Protection [1], Council Directive 2013/59/EURATOM [5], and in Hall EJ [6] and Mettler FA [7] papers.

The questionnaire was self-administered, anonymous and available by personal login and password on a specific web platform. The web database of the University of Pavia automatically captured survey responses. Each question had to be filled in to allow the database admitting the questionnaire. To avoid duplicate entries, the platform was able to prevent a second entry once the user completed the survey.

### Endpoints

The level of knowledge on the topic of ionizing radiation is the primary end-point and was measured as the percentage of correct answers to questions of the knowledge section.

The secondary end points were the perception of physicians about the importance of knowledge in radiation protection, the attendance to previous specific training and the availability of updating in radiation protection. All these were measured by single items present in the questionnaire or combination of them.

### Statistical analyses

Quantitative variables were summarized as means and standard deviation; categorical variables were processed as percentages. The ‘percentage of correct answers’ was computed summarizing the positive answers to the items about the knowledge and comparing to the total of the possible correct answers.

Differences in knowledge between specialist radiologists and the other specialists were investigated. For the quantitative variables unpaired t test with Satterthwaite’s correction for unequal variances if necessary was applied. For qualitative variables chi-square test or Fisher’s exact test as appropriate was used.

The statistical analyses were performed using STATA 12. A *p* value ≤0.05 was considered significant.

## Results

Four hundred nineteen physicians participated to the study with a response rate of 10.5%. These were from doctors specialised in the following macro-areas: general practice (114), specialized medicine (55), neurosciences (29), general surgery (18), specialized surgery (50), diagnostic and laboratory medicine (39), clinical services (41), public health (32). Forty-one doctors were not specialized in any area. The responders included 11.45% family physicians, 64.20% consultants, 19.33% resident physicians, 1.90% academics and 1.67% chief medical officers; in two questionnaires, the occupation was not indicated. The mean age was 44.76 years (± 12.33) and around half of participants (59.43%) had been practicing for more than 10 years. Demographic variables of responders are illustrated in Table 1.

**Table 1:** Demographic variables of responders

	*Frequency*	*Percentage*
*Gender*		
Male	210	50.12
Female	209	49.88
*Years of experience after graduation*		
< 10 years	170	40.57
> 10 years	249	59.43
*Macro-areas of Specialty*		
General practice	114	27.21
Specialized medicine	55	13.13
Neurosciences	29	6.92
General surgery	18	4.30
Specialized surgery	50	11.93
Diagnostic and laboratory medicine	39	9.31
Clinical services	41	9.79
Public health	32	7.64
None	41	9.79
*Occupation*		
Family physician/paediatrician	48	11.45
Consultant	269	64.20
Chief medical officer	7	1.67
Resident	81	19.33
Academic	8	1.90
Other	6	1.43
*Mean age*	44.75 years ±12.33	

Only 202 (48.2%) responders attended formal radiation protection courses during Academic period or professional career. When education was received, it was more commonly in the form of Academic course (76.14%) or Continuing Medical Education (CME) programs (42.62%). 22.91% of doctors indicated to be interested in updating courses on radioprotection. Three hundred sixty-four doctors ordered or performed diagnostic imaging test, mostly in accordance with personal choice (65.9%) or with the specialist dispositions (25.9%). Almost all of this physicians considered radiation protection topics ‘very important’ or ‘quite important’. On the contrary, only 69,45% of all responders believed it.

In average the proportion of correct answers to the questions on ionizing radiation knowledge was 62.29%, slightly greater for the senior doctors with more than 10 years of job experience than for junior doctors with less than 10 years of job experience (63.97% vs 61.14%; *t* = 2.49, *p* = 0.01). The proportion was significantly higher also between radiologists (73.95%) compared to other specialist (61.32%) (*t* = 5.91, *p* < 0.0001). No difference were found between doctors attending specific training and those not attending. Overall, 88.07% of participants answered correctly at almost the half of knowledge questions.

**Fig. 1:**
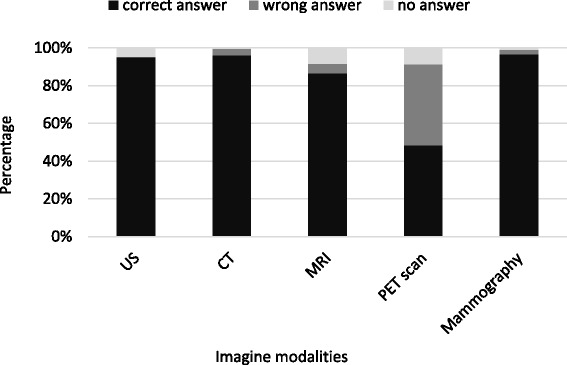
Knowledge about imaging modalities involving ionizing radiation

The most part of physicians rightly indicated that Ultrasound (US) and Magnetic Resonance (MRI) don’t imply use of ionizing radiation (94.75 and 86.63% respectively) (Fig. 1). On the contrary, the item asking to compare doses with equivalent Chest X-ray results in a change of the percentages of correct answers (96.9% for US and 80.91% for MRI).

Only 53.46% identified that Positron Emission Tomography (PET) scan was the only test among 5 (US, CT, MRI, PET and Mammography) implying a radiation emission by the patient after the exam (radioactive material still inside patient), with a statistically significant difference between radiologists and other doctors (60.6% vs 50.4%; p _Fisher’s exact test_ < 0.001).

With the exception of mammography, doctors generally underestimated the radiation doses, and consequently the radiation risk, incurred by the patients undergoing to common radiological investigations. The percentages of correct, underestimated and overestimated answers were summarized in Table 2.

**Table 2:** Percentage distribution of estimations of doses for radiological modalities

*Modalities*	*Underestimated* *n* (%)	*Correct* *n* (%)	*Overestimated* *n* (%)
Abdominal CT	311 (74.22)	108 (25.78)	0 (0)
Column MR	0 (0)	339 (80.91)	80 (19.09)
Abdominal US	0 (0)	406 (96.90)	13 (3.10)
Coronary angiography	362 (86.40)	57 (13.60)	0 (0)
Mammography	21 (5.01)	350 (83.53)	48 (11.46)

**Fig. 2:**
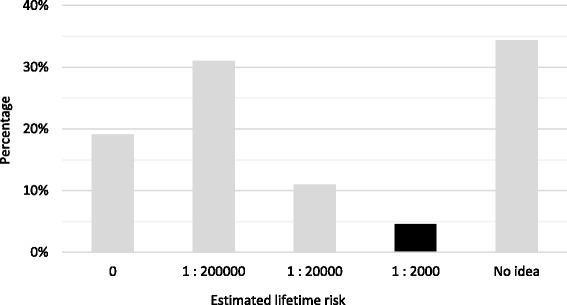
Percentage distribution of increased average risk of malignancy from an abdominal CT (correct answers are marked in *black*)

16.71% of participants properly recognized that almost one radiological procedure involving ionizing radiation increases the malignancy risk. Radiologist correct answers showed a percentage significantly higher than other responders (46.88% vs 14.71%; *p* < 0.001). Only 4.53% correctly estimated the lifetime risk of inducing a fatal cancer from an abdominal CT (Fig. 2), whereas 19.09% (80 respondents) believed that there was no risk. This estimation was statistically different between radiologists and other physicians (12.5% vs 3.88%, p _Fisher_ = 0.048).

**Fig. 3:**
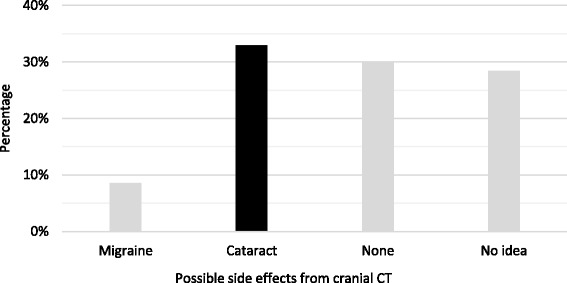
Percentage distribution of eventual side effects consequent to various cranial CT (correct answers are marked in *black*)

Correct choice about possible side effects consequent to more cranial CT was equal to 32.94% (Fig. 3).

In terms of organ sensitivity to radiation exposure, around the half of respondents (46.06%) wrongly thought that thyroid was the more sensitive part, without differences between type of medical specialization. 86.26% of respondents were aware that children were more sensitive than adults and older people to ionizing radiation. Radiologists correctly answer to this topic, with a significant difference with respect to physicians of other specialty (100% vs 88.37%; p _Fisher_ = 0.036).

Focusing on patient’s radiation protection, only 84 responders (20.05%) rightly reported that the patient annual dose limit doesn’t exist, with a relevant difference between radiologists and other specialists (43.75% vs 18.09%, *p* < 0.001). Most of responders (54.89%) correctly indicated that CT accounted for the majority of medical radiation exposure received by the population, but no less than 177 (42.24%) have chosen conventional radiography. Only 17.66% correctly chose natural background as the first source of ionizing radiation for the population (Table 3).

**Table 3:** Percentage distribution of estimations of contribution to collective dose from man-made and natural sources

	*Contribution on collective radiation dose* *n* (%)		
*Ionizing radiation sources*	1	2	3
Medical practices	139 (33.17)	**148 (35.32)**	132 (31.51)
Chernobyl and Fukushima accidents	**203 (48.45)**	125 (29.83)	91 (21.72)
Terrestrial radiation	221 (57.75)	124 (29.59)	**74 (17.66)**

1 = lower contribution, 3 = higher contribution. Correct answer in bold

## Discussion

In our study, the average proportion of correct answers to the questions about ionizing radiation knowledge is 62.29%. The most part of respondents consider very important to be aware about radiation doses of radiological procedures. Less than half of the participants claims to have had previous education on radiation protection and only about 23% is interested in updating radiation knowledge with specific courses.

Overall, respondents show a knowledge on ionizing radiation higher than those reported in previous similar studies (raging 29%–40%) [8–13]. Radiologists appear more knowledgeable than their colleagues, in accordance with evidences from the study performed by Soye et al. [10]. This great knowledge is plausibly in relation with the specific training received during radiology residency.

The frequency of correct answers about US and MRI radiation content is, on average, higher (94.75 and 86.63%, respectively) than the published evidences (ranging 66.1%–96% and 71.4%–87.5%) [9, 10, 13–19]. Surprisingly, the answers to the two questions on the use of ionizing radiation with the same examinations, expressed in different ways give different information. This incongruity highlights the lack of basic knowledge on physic principles involved in the clinical examination.

Only 3.34% of the participants is able to properly evaluate the radiation doses in every single one of the five imaging modalities proposed. In particular, an underestimation of doses for abdominal CT and coronarography (the most risky exams in the list) emerges. These findings are consistent with previous studies [8, 9, 13, 14, 16, 18, 19].

The ratio of the correct answers to the question about the lifetime risk of inducing a fatal cancer from an abdominal CT is in accordance with the evidence from the study of Soylemez et al. [14], performed between Urology residents (3.2%). In contrast, overseas studies [10, 13, 15] report correct estimation of the lifetime risk by more responders (12.5%–27.5%).

The better global performance of our responders compared to those of overseas studies could be due to a more accurate education during medical school, although the most part of doctors declare to have not performed any specific radiation protection training, in accordance with literature.

The present study shows several limits that could influence results. First, the response rate (10.5%) might introduce a selection bias with a overestimation of several evidences. In addition, the web based methodology could lead to under-representation of physicians with limited access to Internet services. Lastly, the questionnaire used to assess physician’s knowledge is not validated nor standardized.

Nevertheless, it is evident that many participants are not enough confident with doses of common imaging tests and with the associated biological effects.

## Conclusions

In conclusion, our findings support the need of raising awareness about risk associated to ionizing radiation procedures. It is imperative to find best modalities and schemes that support a widespread knowledge about radiation protection between physicians and promote the integration of referral guidelines into the clinical practice. In details, we suggest the establishment of a specific course for residents and Continuing Medical Education programs addressed to general practitioners and family pediatricians, who are the first to order imaging tests. The purpose is to minimize unnecessary radiation exposure of the population with improving appropriateness of examinations. It is further to investigate the utility of electronic ad web-based tools as decisional support for doctors in requesting radiological procedures.

## Additional file

Additional file 1: English version of the questionnaire used in the study. (DOCX 17 kb)

## Abbreviations

CME: Continuing Medical Education
CT: Computed Tomography
MRI: Magnetic Resonance Imaging
mSv: millisievert
PET: Positron Emission Tomography
US: Ultrasound
