# Supplementary material for: Are physicians aware enough of patient radiation protection? Results from a survey among physicians of Pavia District– Italy
Source: BMC Health Serv Res. 2017 Jun 14;17:406. doi: 10.1186/s12913-017-2358-1 (PMC5471682; doi:10.1186/s12913-017-2358-1)
Supplement: Additional file 1: — English version of the questionnaire used in the study. (DOCX 17 kb) [file 12913_2017_2358_MOESM1_ESM.docx]

1. What is your impression about radiation protection topics?

very important

quite important

less important

not important

2. Which of the following procedures would you avoid, if equally effective?

CT

MRI

US

None of these procedures is harmful

All procedures are harmful

I don’t know

3. Do the following radiological imaging modalities involve ionizing radiation?

US yes no I don’t know

CT yes no I don’t know

MRI yes no I don’t know

PET yes no I don’t know

Mammography yes no I don’t know

4. Do the following radiological imaging modalities result in emitting radiation from the patient after execution?

US yes no I don’t know

CT yes no I don’t know

MRI yes no I don’t know

PET yes no I don’t know

Mammography yes no I don’t know

5. For each of the following procedures, state the patient dose in term of equivalent number of chest X-ray

Abdominal CT 0 1-50 51-300 >300

Column MRI 0 1-50 51-300 >300

Abdominal US 0 1-50 51-300 >300

Coronary angiography 0 1-50 51-300 >300

Mammography 0 1-50 51-300 >300

6. Could the exposure to a single radiological procedure increase the cancer risk?

Yes

Yes, if doses are elevated

No

I don’t know

7. Could repeated exposure to radiological procedures increase the cancer risk?

Yes

No

No, if there is a timeframe between procedures

I don’t know

8. Repeated brain CT could result in:

Headache

Cataract

None

I don’t know

9. What is the estimated risk of cancer for a patient undergoing abdomen CT?

0

1/200.000

1/20.000

1/2.000

I don’t know

10. Which of the following are accepted as the most radiosensitive organs?

Thyroid

Lung

Skin

Bone

Bone marrow

Brain

I don’t know

11. Which of the following is the most radiosensitive age group?

Older people

Adults

Children

All ages are equally sensitive

I don’t know

12. What is the annual dose a patient is allowed to receive (20 mSv/year is the limit of radiation dose that a worker is allowed to receive in one year)?

50 mSv

20 mSv

10 mSv

Unlimited

I don’t know

13. Italian population is exposed to several ionizing radiation sources. Give a value from 1 to 3 to the following sources based on their contribution to the Italian collective dose (1= lower contribution, 3= higher contribution)

Medical radiation

Chernobyl and Fukushima accidents

Terrestrial radiation

14. Which of the following modalities is accountable for the highest collective dose for Italian population?

Conventional X-ray

CT

Ultrasound

MRI

I don’t know

15. In performing your professional practice, could you prescribe or perform diagnostic imaging test?

Yes/No

16. If your answer to question 15 is yes, do you believe that knowledge about radiation doses is important?

very important

quite important

less important

not important

17. If your answer to question 15 is yes, which of the following mainly sway radiological prescription?

Patients’ or relatives’ choice

Specialist dispositions

Personal choice

Other, specify ______________

18. During Academic period or professional career, have you received any specific training regarding radiation protection?

Yes

No

I don’t remember

19. If your answer to question 18 is yes, please state type of training

Academic lecture yes/no/don’t remember

Workshop yes/no/don’t remember

Continuing Medical Education programme yes/no/don’t remember

Advanced training/Master yes/no/don’t remember

Other yes/no/don’t remember

20. Would you be interested in updating courses on radiation protection?

Yes/No

21. Year of birth

22. Gender

Male Female

23. What is your occupation?

family physician

family paediatrician

consultant

resident physician

academic

chief medical officer

other, specify ______________

24. Principal Medical specialty achieved

25. Other Medical specialty achieved

26. What is the length of your experience after graduation?

<5 5-10 11-20 >20
